# Granulomatous Hepatitis Treated With Certolizumab Pegol

**DOI:** 10.14309/crj.0000000000001310

**Published:** 2024-03-28

**Authors:** Kevin Brittan, Alexandra Fiedler, Kyle Scholten, Busara Songtanin, Shane Manatsathit

**Affiliations:** 1 Department of Internal Medicine, College of Medicine, University of Nebraska Medical Center, Omaha, NE; 2 Department of Internal Medicine, Texas Tech University Health Sciences Center, Lubbock, TX; 3 Division of Gastroenterology and Hepatology, Department of Internal Medicine, University of Nebraska Medical Center, Omaha, NE

**Keywords:** Hepatology, granulomatous hepatitis, certolizumab, anti-drug antibodies

## Abstract

Idiopathic granulomatous hepatitis is a rare condition characterized by hepatic granulomas with constitutional symptoms such as recurrent fevers, myalgias, and hepatosplenomegaly in the absence of infection or inflammatory disorder. Typical treatment and course of this disease consist of a course of steroids with rapid symptom resolution. However, symptoms may recur when steroids are tapered. In these circumstances, azathioprine, methotrexate, infliximab, and adalimumab have demonstrated good response. In this case, we present a patient who developed antidrug antibodies to infliximab and adalimumab and was the first documented case of this disease to be treated with certolizumab pegol. Our case highlights the novel efficacy of certolizumab pegol for idiopathic granulomatous hepatitis and its role in treating idiopathic granulomatous hepatitis with antidrug antibodies.

## INTRODUCTION

Idiopathic granulomatous hepatitis accounts for 10%–35% of all granulomatous hepatitis.^1-3^ It is characterized by febrile illness, myalgias, hepatosplenomegaly, and arthralgias in patients with hepatic granulomas and no other causative etiology. Typically, prednisone is initiated and tapered over 4 to 8 weeks with rapid symptomatic improvement. Liver enzymes tend to remain elevated for weeks to months, and granulomas will slowly resolve with steroids. In refractory cases or recurrent flares upon steroid tapering, patients are managed with nonsteroidal agents. Azathioprine, methotrexate, infliximab, and adalimumab have been previously validated as effective therapies.^1,2^ The efficacy of these biological agents can wane over time as patients develop anti-drug antibodies (ADAs). We present a 37-year-old woman with idiopathic granulomatous hepatitis who, although developed ADAs to both infliximab and adalimumab, was successfully treated with certolizumab pegol.

## CASE REPORT

A 37-year-old woman with a history of erythema nodosum presents for acute worsening of intermittent abdominal pain and fevers. The patient had similar symptoms at age 23 with fevers, abdominal pain, and a liver mass on abdominal computed tomography that resolved with a short course of steroids. A liver biopsy was obtained for further evaluation due to ongoing symptoms demonstrating granulomatous necrotizing inflammation, and the subsequent fungal stain was negative. The patient's symptoms slowly resolved with no further interventions at that time. Over the next 14 years, she developed recurrent fevers, abdominal pain, and arthralgias approximately every 2 years that self-resolved. An esophagogastroduodenoscopy and colonoscopy were performed during this time with no abnormalities noted.

**Figure 1.**
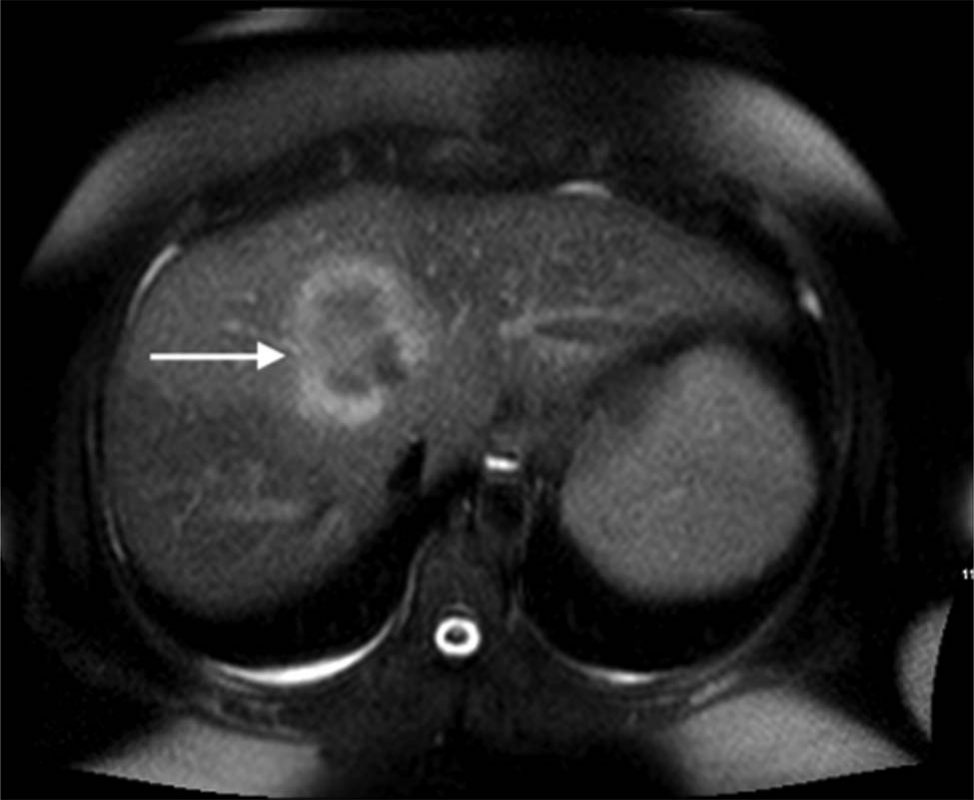
T2 FLAIR-weighted magnetic resonance imaging demonstrating pretreatment 61 by 49 mm hepatic granuloma. FLAIR, fluid-attenuated inversion recovery.

At re-presentation, the patient reported 1 month of constant abdominal pain, fevers, and recurrent nonbloody, nonbilious emesis. The patient denied any current medication use. An abdominal and pelvic computed tomography scan and subsequent magnetic resonance imaging (MRI) demonstrated the presence of a liver mass measuring 61 by 49 mm in the right central lobe (Figure 1). Biopsy of the granuloma demonstrated necrotic granulomatous inflammation similar to the prior biopsy. Infectious etiologies were assessed with negative work-up for brucella, q-fever, tuberculosis, Lyme disease, histoplasmosis, human immunodeficiency virus, and viral hepatitis. Rheumatologic evaluation consisted of negative antinuclear antibodies, antineutrophilic cytoplasmic antibody, anti-mitochondrial antibodies, and chest x-ray with absent hilar adenopathy. A diagnosis of idiopathic granulomatous hepatitis was made, and the patient had an initial resolution of symptoms with a course of 40 mg of prednisone that was tapered over 2 months. Over the next year, she had 2 reoccurrences of symptoms with a repeat MRI showing a decrease in the size of the original granuloma to 12 mm, but an additional 6 mm granuloma was identified. Despite additional courses of prednisone, the patient continually re-presented with symptoms upon steroid tapering.

**Figure 2.**
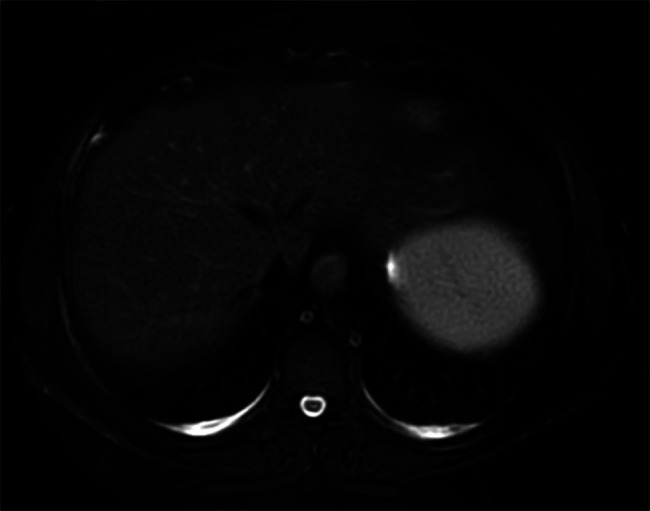
T2 FLAIR-weighted magnetic resonance imaging demonstrating resolution of hepatic granuloma after treatment with certolizumab pegol. FLAIR, fluid-attenuated inversion recovery.

To evaluate additional therapy options, thiopurine methyltransferase levels were measured and low; therefore, azathioprine was not initiated. Instead, the patient was started on 50 mg of adalimumab every 2 weeks and 5 mg of methotrexate. After an initial improvement in symptoms, the patient failed to respond to adalimumab after 1 year. Further evaluation showed undetectable levels of adalimumab in the presence of ADAs. Methotrexate was optimized to 25 mg subcutaneously, and infliximab was trialed at 5 mg/kg. Similarly, she developed ADAs to infliximab after a year and required chronic steroids to maintain remission. Ultimately, the patient started certolizumab pegol at an initial dose of 400 mg followed by 200 mg every 2 weeks for maintenance and 25 mg of methotrexate subcutaneously for further immunosuppression in attempts to prevent the development of ADAs while minimizing future steroid use. Symptoms resolved, and no granulomas were identified on subsequent MRI obtained 1 year after prior imaging (Figure 2). Steroids were able to be tapered to 2.5 mg of prednisone daily, and to date, the patient has successfully remained in remission on certolizumab pegol.

## DISCUSSION

Idiopathic granulomatous hepatitis comprises up to a third of granulomatous hepatitis cases.^3^ It is traditionally managed with steroids and methotrexate, azathioprine, or anti-tumor necrosis factor (TNF) α antagonists in chronic cases.^1,2^ In this case, the patient developed multiple antidrug antibodies (ADAs) in the setting of refractory disease. This patient's lack of response to proven anti-TNF α agents and adverse side effects of chronic steroid use obligated further utilization of novel steroid-sparing agents. This case is the first documented use of certolizumab pegol for the management of idiopathic granulomatous hepatitis.

Certolizumab pegol offers unique advantages compared with other anti-TNF α agents. The PEGylated Fab fragment increases the half-life to approximately 2 weeks.^4^ Moreover, the monovalent Fab fragment can easily disassociate in drug-ADA complexes.^5^ The combination of these structural alterations confers additional resistance to ADAs. Prior studies on patients with rheumatoid arthritis have demonstrated approximately 37%–65% of patients will develop ADAs to cetrolizumab.^6^ Despite the presence of ADAs, high levels of certolizumab were still present in most of these patients.^5^ Therefore, it remains a therapeutic option despite the development of antibodies. Owing to this unique profile, certolizumab pegol was an advantageous treatment option for our patient with prior failure to multiple biologic agents due to the development of ADAs.

This case documents the novel use of certolizumab pegol for the successful treatment of idiopathic granulomatous hepatitis. In patients with failure of anti-TNF α therapies secondary to ADAs, certolizumab pegol should be considered as a therapeutic option.

## DISCLOSURES

Author contributions: All authors made substantial contributions to the conception and drafting of this article. All authors edited and revised this work and have agreed on its accuracy. K. Brittan is the article guarantor.

Financial disclosure: None to report.

Informed consent was obtained for this manuscript.
